# Comparison of growth factor adsorbed scaffold and conventional scaffold with growth factor supplemented media for primary human articular chondrocyte 3D culture

**DOI:** 10.1186/s12896-014-0108-6

**Published:** 2014-12-28

**Authors:** Jeerawan Klangjorhor, Thanyaluck Phitak, Dumnoensun Pruksakorn, Peraphan Pothacharoen, Prachya Kongtawelert

**Affiliations:** Thailand Excellence Center for Tissue Engineering and Stem Cells, Faculty of Medicine, Chiang Mai University, Intravarorot Road, Sripoom, Chiang Mai, 50200 Thailand; Department of Orthopedics, Musculoskeletal Research Laboratory, Faculty of Medicine, Chiang Mai University, Intravarorot Road, Sripoom, Chiang Mai, 50200 Thailand

**Keywords:** Gelatin scaffold, Human articular chondrocytes, Extracellular matrix, HA, TGF-β3, Cartilage tissue engineering

## Abstract

**Background:**

Cartilage tissue engineering offers new strategies in repairing damaged cartilage. Scaffolds have been used for the *in vitro* and *in vivo* procedures for this application, which demonstrates the compatible biological and physical properties that mimic natural tissues. Several types of scaffolds were used and had different effects on cell functions. The study was designed to develop a functional gelatin scaffold by adsorption of hyaluronan (HA) and the transforming growth factor β3 (TGF-β3) in a commercially available gelatin scaffold.

**Results:**

The biological properties of human articular chondrocytes were investigated during a 21-day cultivation embedded in either HA + TGF-β3 adsorbed scaffolds or the conventional supplemented method. The rising of proliferation of chondrocytes embedded in adsorbed scaffolds was observed at day 17 and 21 of cultivation (1.27 and 1.28 fold, respectively). The chondrogenic gene expression of the chondrocytes embedded in HA + TGF-β3 adsorbed scaffolds significantly increased: *SOX-9* (1.65 fold), *ACAN* (7.65 fold) and *COL2A1* (1.83 fold). Remarkably, over the 21 days of cultivation, HA + TGF-β3 adsorbed scaffolds promoted the extracellular matrix molecules production with higher accumulation of HA (1.2 fold), collagen (1.42 fold) and uronic acid (1.41 fold). Moreover, the cell population and extracellular matrix production, which were examined by a histological analysis and a scanning electron microscope, were correlated with the biochemical analysis.

**Conclusion:**

A small amount of HA and TGF-β3 initially adsorbed in the scaffolds (70 μg and 10 ng, respectively) was consumed over the 21-day cultivation. The HA + TGF-β3 adsorbed gelatin scaffold is effective and more suitable than the conventional supplemented method for the *in vitro* assessment of human chondrocyte 3D culture.

## Background

Chondral defects can lead to long term cartilage and bone degeneration, if they are not diagnosed and treated. Chondrocytes have a limited capacity for self-repair. Damage frequently results in developmental abnormalities or age related degeneration, such as osteoarthritis [1]. A current approach for regenerative treatment is Autologous Chondrocyte Implantation (ACI) [2-4]; however, this technique is limited by the number of cells available, size of the defects, and the need to maintain fully-differentiated chondrocyte functions.

Tissue engineering has been introduced as a treatment for cartilage damage because of its potential to enhance and improve the efficacy of ACI. It has been developed for restoring, maintaining, and enhancing tissue or organ function [5-8]; moreover, culturing isolated cells on biocompatible and biodegradable scaffolds *ex vivo* can generate tissue for *in vivo* implantation [9].

A variety of biodegradable polymers have been explored for cartilage repair, such as collagen sponges [10-12], agarose [13,14], chondroitin sulfate [11,15,16] and silk [17]. Scaffolds provide a three-dimensional (3D) environment that promotes chondrogenesis, prevents dedifferentiation, and helps in the recovery of the fully-differentiated chondrocyte phenotype, which is lost in a two-dimensional (2D) culture [18-21]. Previously, it has been reported that a gelatin-based scaffold (SPONGOSTAN® Standard, Johnson & Johnson) is suitable for use as an *in vitro* model for chondrocyte 3D culture [1,22]. There are several factors that affect the quality of cartilage generated in tissue culture, including the type of scaffold material used, the quality of the chondrocytes, and the culture media including growth factors [1]. In hyaline cartilage, hyaluronan (HA) plays important roles in the skeletal network and provides stability to the extracellular matrix molecules assembling through interaction with other matrix components and chondrocytes. HA promotes chondrocyte proliferation, morphology, and migration, and it provides a controlled environment suitable for cell growth and tissue formation [1,8]. The application of HA for cartilage tissue engineering has been studied. Either adding HA into the culture media or exogenous HA-treated scaffolds has been reported to provide positive effects for chondrocyte growth and differentiation [1,8,12]. In conventional techniques, chondrocytes/scaffolds are cultured in chondrogenic media supplemented with growth factors. TGF-β3 is one of the growth factors studied, and it can induce the expression of anabolic chondrogenic gene markers such as α1- Collagen type II (*COL2A1*) and cartilage oligomeric matrix protein (*COMP*), leading to induction of extracellular matrix production [23,24]. However, large amounts of TGF-β3 are needed as frequent media changes require constant replacement.

The scaffold chondrogenic growth factors and biochemical stimuli have been shown to be important factors for cartilage tissue engineering. Therefore, this study sought to determine whether a HA/TGF-β3 physically immobilized scaffold could retain or enhance chondrogenic properties required for cartilage transplantation better than the scaffold with soluble growth factors. The chondrogenic properties were investigated with primary human articular chondrocyte culture by examining cell proliferation and extracellular matrix production as well as by using scanning electron microscopy and a histological examination. The expression of anabolic chondrogenic genes was also measured over a period of a 21-day cultivation.

## Results

### Amount of TGF-β3 and HA in cultivation

The chondrogenic media for the non-adsorbed scaffold control was supplemented with the same amounts of TGF-β3 (10 ng/ml) and HA (70 μg/ml) as in the conventional method. During the cultivation, the medium was changed every 3 days. Thus, the total amounts of TGF-β3 and HA used in this soluble control group were 60 ng and 420 μg respectively (Table 1). This contrasted with the adsorbed scaffold group in which the total amounts of TGF-β3 and HA used were only that adsorbed on the scaffold. By using an adsorbed scaffold, the use of TGF-β3 and HA was reduced by more than 80%. In order to investigate the release profile of HA and TGF-β3 adsorbed to the scaffold, the HA and TGF-β3 were quantified in the culture media of controlled incubations (scaffold control). Interestingly, more than 90% of the TGF-β3 and HA remained on the scaffold after 21 days. The data showed that the gelatin scaffold could adsorb HA and TGF-β3, so that the culture showed only a slow rate of release.

**Table 1:** **Amount of biomolecules used in cultivation and quantification of adsorbed HA and TGF-β3 within gelatin scaffolds at day 21 of cultivation**

**Name of experimental group**	**Amount of biomolecules used in curtivation**		**Amount of biomolecules remained in scaffolds at day 21**			
	**TGF-β3 (ng)**	**HA (μg)**	**TGF-β3 (ng)**		**HA (μg)**	
PBS control	-	-	-		-	
Soluble control (X 6 times of culture media exchange)	60.0	420.0	-		-	
HA adsorbed scaffold	-	70.0		-	67.24 ± 0.37	(96.06 ± 0.53%)
TGFβ3 adsorbed scaffold	10.0	-	9.48 ± 0.098	(94.8 ± 0.98%)	-	
HA + TGFβ3 adsorbed scaffold	10.0	70.0	9.71 ± 0.11	(97.1 ± 1.10%)	66.12 ± 0.23	(94.46 ± 0.32%)

### Cell proliferation on gelatin scaffolds

**Figure 1:**
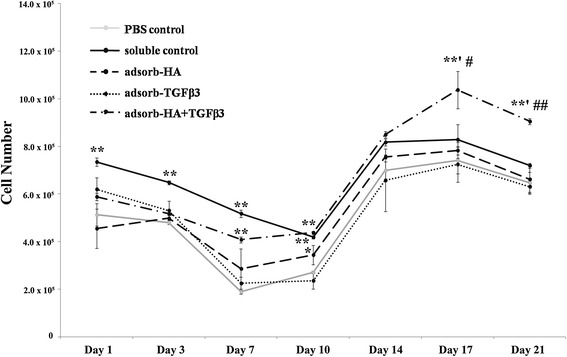
**Cell proliferation analysis of chondrocytes cultured in gelatin scaffolds.** Cell proliferation was examined on days 1, 3, 7, 10, 14, 17 and 21 of cultivation. This data represents three separate experiments (*n* = 3). *, # *p*-values are < 0.05; **, ## p-values are < 0.01; * is a significant amount compared to the PBS control; # is a significant amount compared to the soluble control.

The cell viability and proliferation of chondrocytes-cultured scaffolds were determined on days 1, 3, 7, 10, 14, 17 and 21, by the Alamar Blue (resazurin based) assay by comparing the groups of non-adsorbed scaffolds (PBS control), single factor adsorbed scaffolds (HA adsorbed and TGF-β3 adsorbed scaffolds), combined factors adsorbed scaffolds (HA + TGF-β3 adsorbed scaffolds) and non-adsorbed scaffolds in medium supplemented with soluble HA and TGF-β3 (soluble control). The results showed that the number of cells on days 1 to 10 showed some decline in all cultures and were considered to be a lag phase, whereas days 10 to 17 showed rapid growth, suggesting an exponential or logarithmic phase (Figure 1). The highest survival rate of cells in the cell-seeded soluble control scaffolds was observed earlier on, during days 1 to 7. After day 10, the HA + TGF-β3 adsorbed scaffolds showed higher cell proliferation when compared with soluble control on days 17 and 21 (*p* < 0.05 and *p* < 0.01 respectively). Only HA + TGF-β3 adsorbed scaffolds had significantly increased cell proliferation more than the PBS control on days 17 (1.34 fold, *p* < 0.01) and 21 (1.33 fold, *p* < 0.01). Moreover, increased cell proliferation was observed in cells seeded through the adsorption of HA, HA + TGF-β3 adsorbed scaffolds, and soluble control scaffolds, but not in TGF-β3 adsorbed scaffolds. This data suggested that HA could maintain cell proliferation, and the combination of HA and TGF-β3 was efficient for enhanced cell proliferation.

### Expression of cartilage-specific extracellular matrix biomolecules

The chondrogenic activity of the chondrocyte-seeded scaffolds was assessed by determining the production of a hyaline-like extracellular matrix including HA and s-GAG. The cell-free scaffolds were cultured and determined for HA release in order to compare them to the HA production of cell-seeded scaffolds (as described in the Methods section).

**Figure 2:**
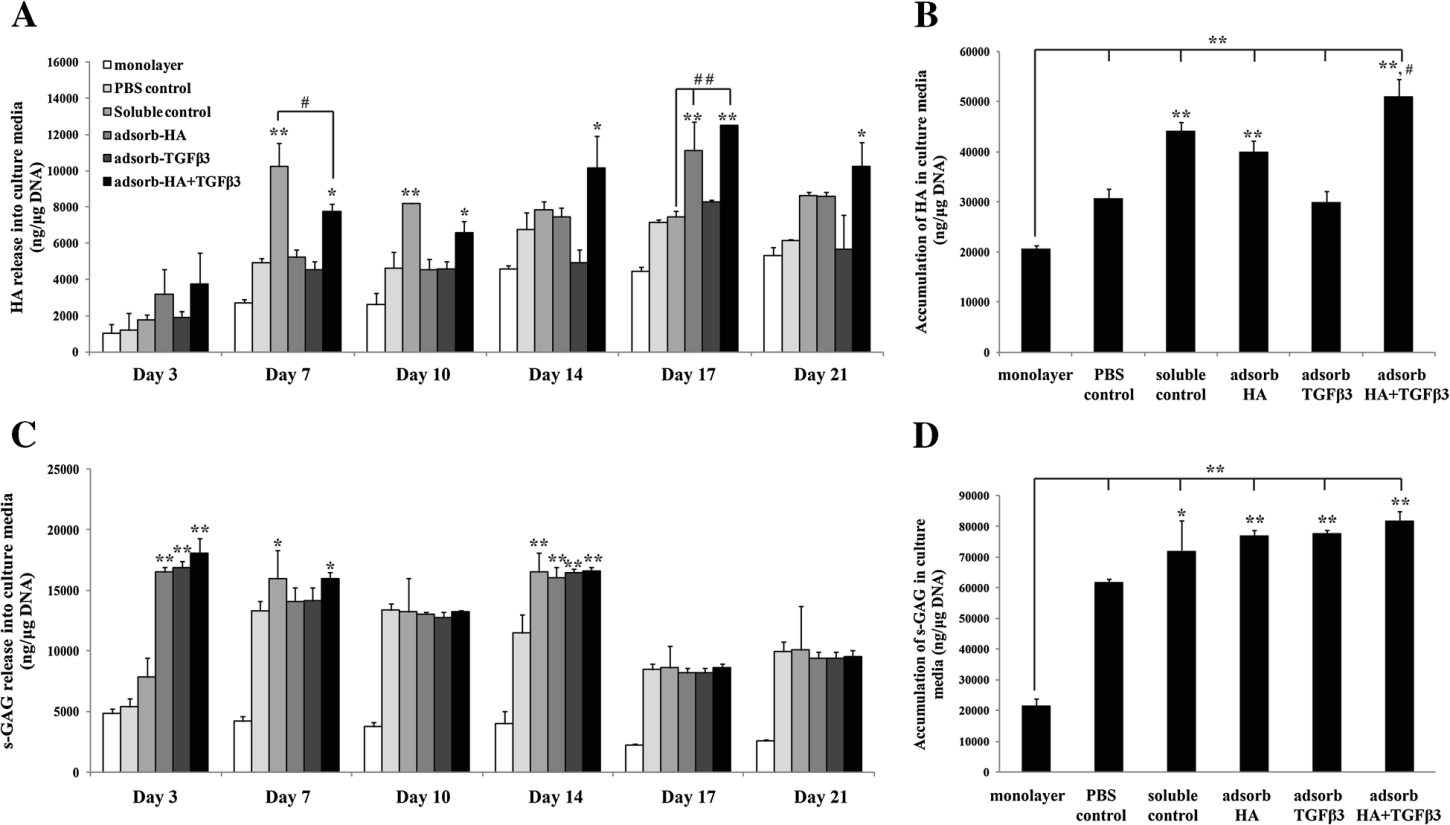
**Glycosaminoglycans concentration in chondrocyte-seeded adsorbed scaffold culture media.** Human articular chondrocytes were cultured for 21 days, and Glycosaminoglycans, which were released into the culture media, were measured at 3-day intervals **(A)**. HA accumulation in the culture media **(B)**. s-GAG released into the culture media at 3-day intervals **(C)**. s-GAG accumulation in the culture media **(D)** were measured (*n* = 3). *, # *p*-values are < 0.05; **, ## p-values are < 0.01; * is a significant amount compared to the PBS control; # is a significant amount compared to the soluble control.

For each 3-day interval, the chondrocyte-seeded gelatin scaffolds showed higher levels of HA production and HA release into the media than monolayer cultures (Figure 2A). At days 7 to 21, the HA + TGF-β3 adsorbed scaffolds showed significantly increased HA release when compared with monolayer cultures and PBS control. The soluble control showed a significantly higher HA release than that of HA + TGF-β3 adsorbed scaffolds at day 7 (*p* = 0.032) and day 10 (*p* = 0.103). However in the late period of the cultivation, HA release in the soluble control was significantly lower than that of HA + TGF-β3 adsorbed scaffolds on day 17 (*p* = 0.001). The HA in culture media was more significantly increased (Figure 2B) in cell-seeded gelatin scaffolds than in monolayer cultures. In the HA + TGF-β3 adsorbed scaffolds, the HA was significantly higher than both PBS control (1.65 fold and *p* < 0.01) and soluble control scaffolds (1.15 fold and *p* = 0.021). The production and release of s-GAG was determined (Figures 2C and 2D) for each of the 3-day intervals. The levels of s-GAG for chondrocyte-seeded soluble control and all types of adsorbed scaffolds were significantly higher than that of monolayer cultures, although there was no difference in the s-GAG released on each day amongst these groups (Figure 2C). However, the levels of s-GAG which accumulated (Figure 2D) in adsorbed scaffolds were slightly higher than those in soluble controls. Of important note is the observation that the HA + TGF-β3 adsorbed scaffolds showed the highest accumulation (81,967 ± 2,894 ng/μg DNA content.

**Figure 3:**
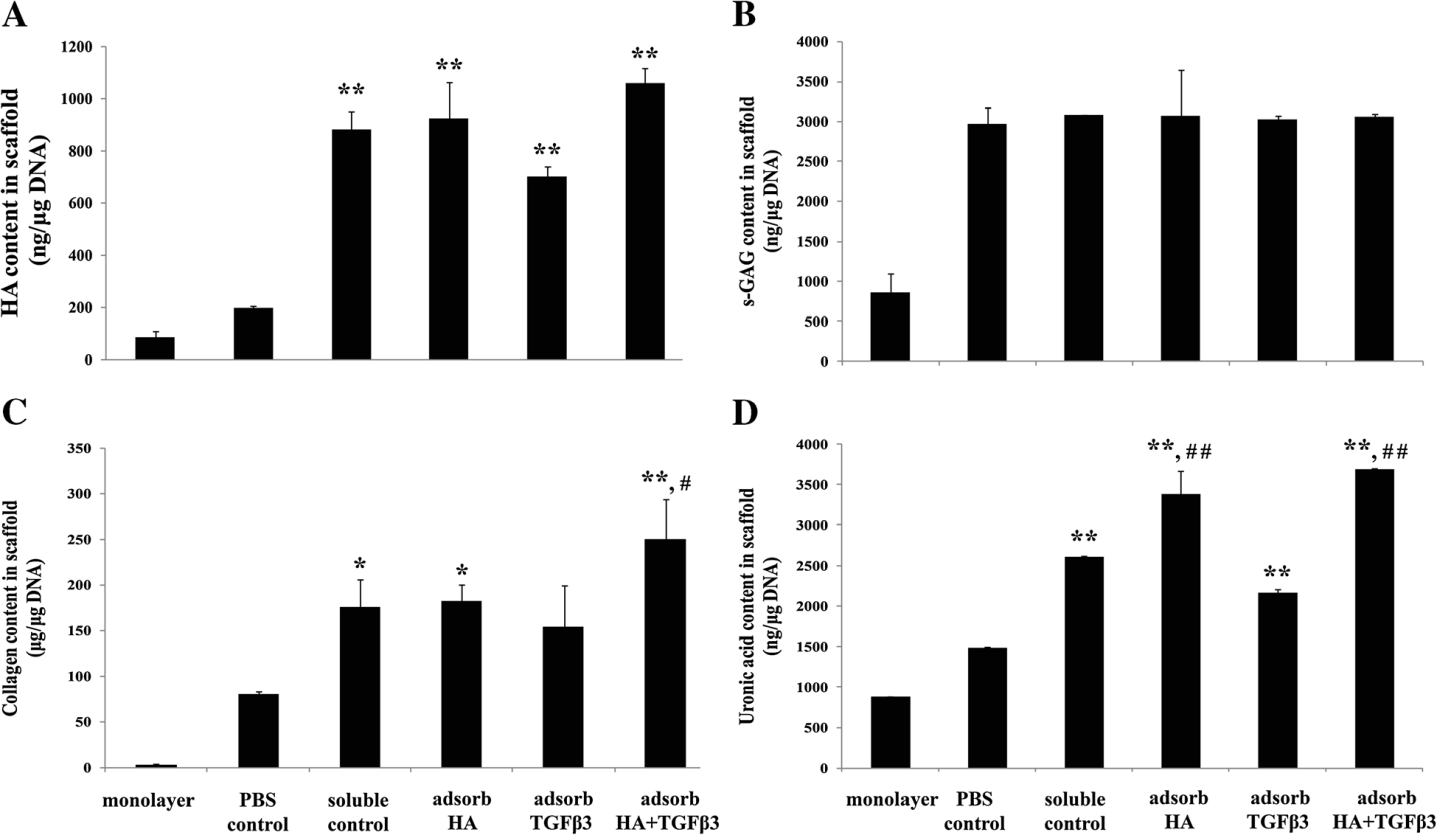
**Cartilage-specific extracellular matrix production in monolayer cultures and gelatin scaffolds.** Human articular chondrocytes were cultured on scaffolds for 21 days and then digested with papain. The papain digests were assayed for cartilage extracellular matrix components including HA **(A)**, s-GAG **(B)**, collagen **(C)** and uronic acid **(D)** content. *, # *p*-values are < 0.05; **, ## p-values are < 0.01; * is a significant amount compared to the PBS control; # is a significant amount compared to the soluble control.

To support this analysis, the process of quantifying the cartilage specific extracellular matrix in cultured scaffolds was performed, including the amounts of HA, s-GAG, uronic acid, and collagen content (Figure 3). After the 21-day cultivation, the levels of ECM production of chondrocytes cultured in scaffolds were significantly higher than those found in the monolayer cultures. Soluble control and all adsorbed scaffolds showed significantly increased amounts of HA in scaffolds compared to the amounts found in PBS control (Figure 3A), and the amounts found in HA + TGF-β3 adsorbed scaffolds were not different from those in the soluble control. The matrix s-GAG content was more abundant in all scaffolds than in monolayer cultures, although the values were not different between the various types of scaffolds (Figure 3B).

The amounts of collagen in soluble control and adsorbed scaffolds were higher than in the PBS control scaffolds (Figure 3C), and the HA + TGF-β3 adsorbed scaffolds showed the highest amounts and were significantly higher than those found in the soluble control (1.42 fold and *p* = 0.046).

At the end of the 21-day cultivation, the amount of uronic acid in the matrix was measured. It was found that in soluble control and adsorbed scaffolds, it was significantly higher than in the PBS control (*p* < 0.01 for all groups (Figure 3D)). In addition, HA and HA + TGF-β3 adsorbed scaffolds had significantly increased levels compared to the level of the soluble control (1.30 and 1.41 fold respectively and *p* < 0.01 in both scaffolds). This extracellular matrix production data suggested that the combination of HA and TGF-β3 adsorbed on the gelatin scaffolds had improved efficiency in enhancing cartilage-specific extracellular matrix production when compared to the method of adsorbing HA or TGF-β3 alone.

### Expression of anabolic chondrogenic mRNA profile of cells on scaffolds

**Figure 4:**
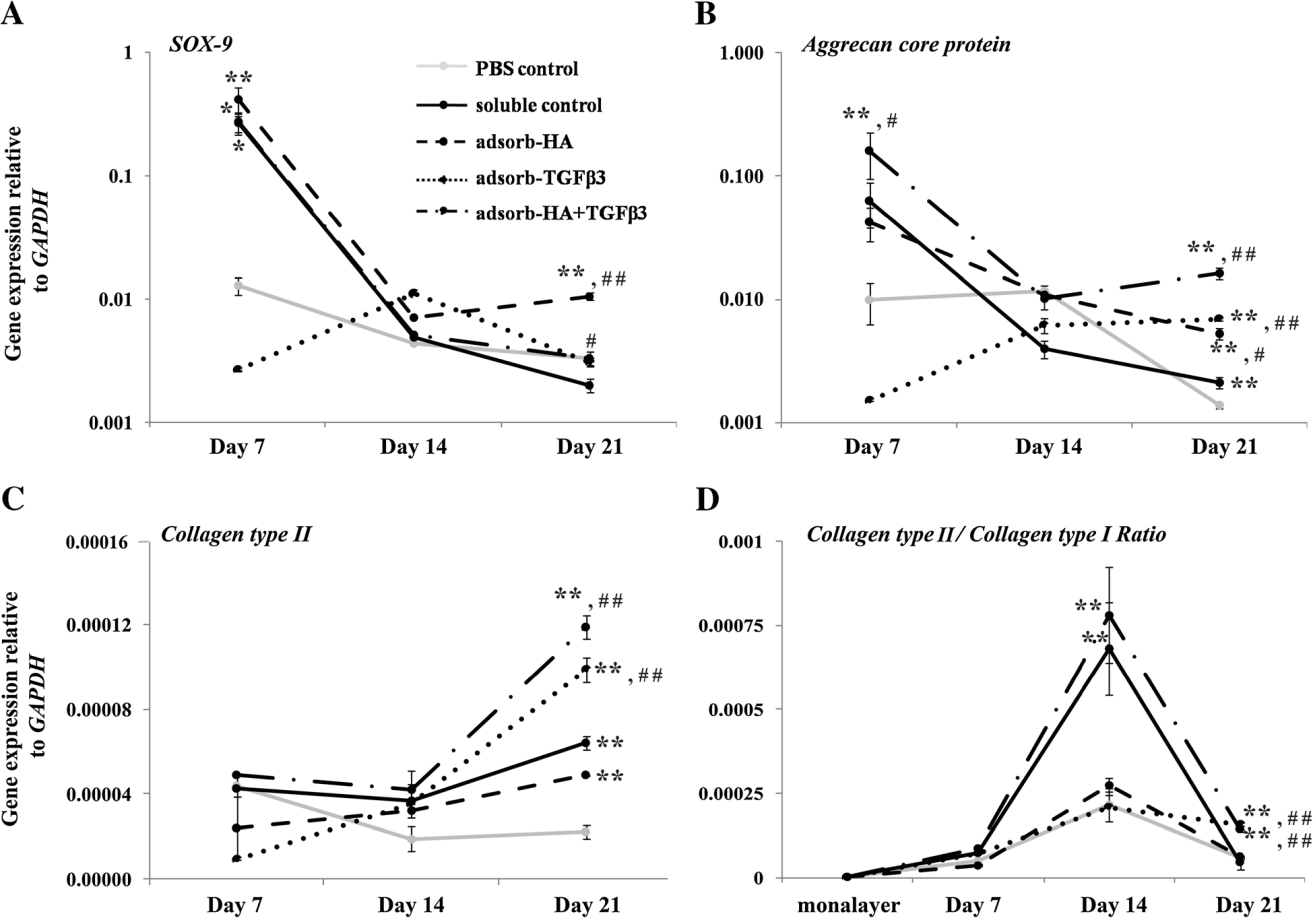
**Quantitative real-time RT-PCR analysis of chondrocytes cultured in gelatin scaffolds.** The results represent the chondrogenic specific gene markers, including SOX-9 **(A)**, Aggrecan core protein **(B)**, Collagen type II (C) and the ratio of Collagen type II/Collagen type I **(D)**. This data represents three separate experiments (*n* = 3). *, # *p*-values are < 0.05; **, ## p-values are < 0.01; * is a significant amount compared to the PBS control; # is a significant amount compared to the soluble control.

To test further whether the adsorbed scaffolds demonstrated better potency of chondrogenic properties than non-adsorbed scaffolds, the chondrogenic gene expression was quantified, including SOX-9 (*SOX9*), Aggrecan core protein (*ACAN*), Collagen type II (*COL2A1*) and Collagen type I (*COL1A2*) by real-time RT-PCR. The mRNA expression levels were monitored on day 7 of the 21-day cultivation. *SOX9* expression showed a fall in all cultures from days 7 to 14 during the rapid proliferation of the cells and remained decreased until the end of the cultivation. However, the expression in HA, HA + TGF-β3 adsorbed and soluble control scaffolds on day 7 was significantly higher than in the PBS control scaffolds (*p* < 0.01, *p* < 0.05 and *p* < 0.05 respectively) (Figure 4A). On day 14, the *SOX9* expression in all adsorbed scaffolds and soluble control scaffolds was higher than in the PBS control, but this was not significant. However, at day 21, the HA and HA + TGF-β3 adsorbed scaffolds showed significantly increased *SOX9* expression when compared to the expression in the soluble control (*p* < 0.01 and *p* = 0.046). Aggrecan (*ACAN*) expression also paralleled some of the changes in *SOX9* (Figure 4B), but comparisons showed that the HA + TGF-β3 adsorbed scaffolds had a marked-up regulation of *ACAN* expression at day 7 and day 21 when compared to that of the PBS control (*p* < 0.01 and *p* < 0.01) and the soluble control (*p* = 0.042 and *p* < 0.01). This correlated with the *SOX9* expression at these same time intervals. On day 21, the level of *ACAN* expression in all adsorbed scaffolds and soluble control scaffolds was significantly higher than in the PBS control (*p* < 0.01 for all groups). As for Collagen type II (*COL2A1*), the expression did not follow the pattern shown for *SOX9* and *ACAN*, and there was no decrease shown from days 7 to 14. At day 21, the expression of *COL2A1* in the chondrocyte-seeded adsorbed scaffolds and soluble control was significantly higher than in the PBS control (*p* < 0.01 for all groups (Figure 4C)). Only the expression in the TGF-β3 and HA + TGF-β3 adsorbed scaffolds were significantly higher than in the soluble control on day 21 (*p* < 0.01 in both groups). All of the adsorbed scaffolds showed an increase of *COL2A1* expression after the 7-day interval while that of the PBS control decreased.

Furthermore, an investigation was made into the ratio of Collagen type II (*COL2A1*) to Collagen type I (*COL1A2*) gene expression as a measure of chondrocyte phenotype (Figure 4D). All gelatin scaffolds showed higher ratios of *COL2A1* to *COL1A2* than in monolayer cultures. The ratio was at its highest on day 14 and decreased at day 21. The ratio measured in chondrocyte-seeded HA + TGF-β3 adsorbed scaffolds was significantly higher than that of the PBS control and soluble control at both day 14 and 21.

### Histology examination of chondrocytes cultured on scaffolds

**Figure 5:**
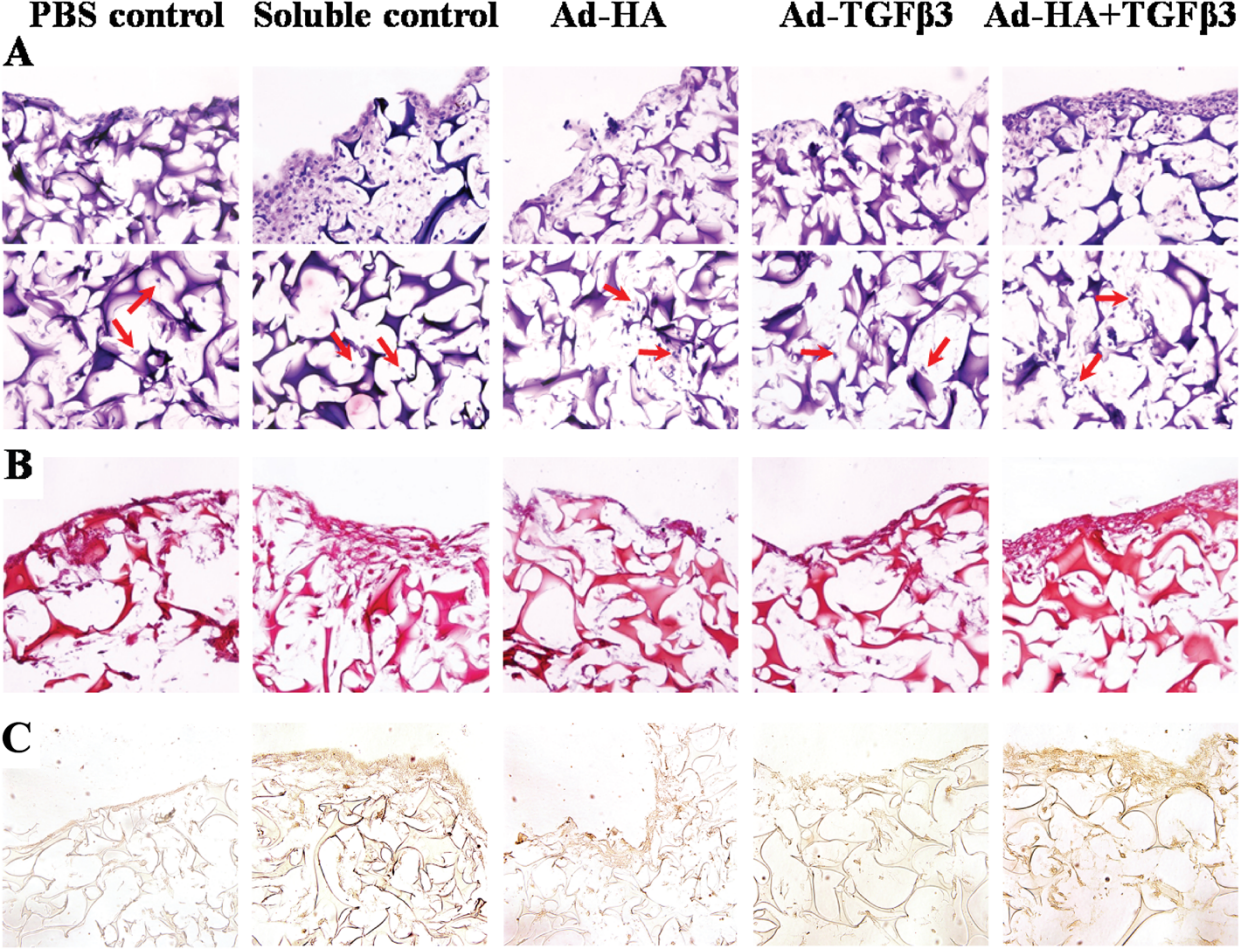
**Histological examination of chondrocyte-seeded gelatin scaffold cultures after 21 days.** Hematoxylin and Eosin staining **(A)** for cell morphology and distribution; arrow indicates cells. Safranin O staining **(B)** for glycosaminoglycans production and immunohistochemistry for collagen type II production **(C)**.

The cell penetration and morphology of chondrocyte-seeded scaffolds by hematoxylin & eosin staining was determined in this study (Figure 5A). The HA + TGF-β3 adsorbed scaffolds showed plentiful cell penetration and growth in both the edge (higher panel) and porous areas (lower panel). The HA adsorbed scaffolds showed less cell distribution than that of the HA + TGF-β3 adsorbed scaffolds. The other scaffold types, including the PBS control, soluble control, and TGF-β3 adsorbed scaffolds, showed less cell growth and distribution within the scaffolds.

To determine the extracellular matrix production, scaffolds were stained with Safranin O for glycosaminoglycans and with immunohistochemistry for Collagen type II (Figures 5B and 5C). The results showed that the HA + TGF-β3 adsorbed scaffolds had higher amounts of Safranin O staining when compared to the amounts in the other scaffolds with the same observation of Collagen type II intensity (shown in brown).

The results showed that the HA + TGF-β3 adsorbed scaffolds were the best at enhancing cell proliferation and extracellular matrix production.

### Scanning electron microscopic examination

**Figure 6:**
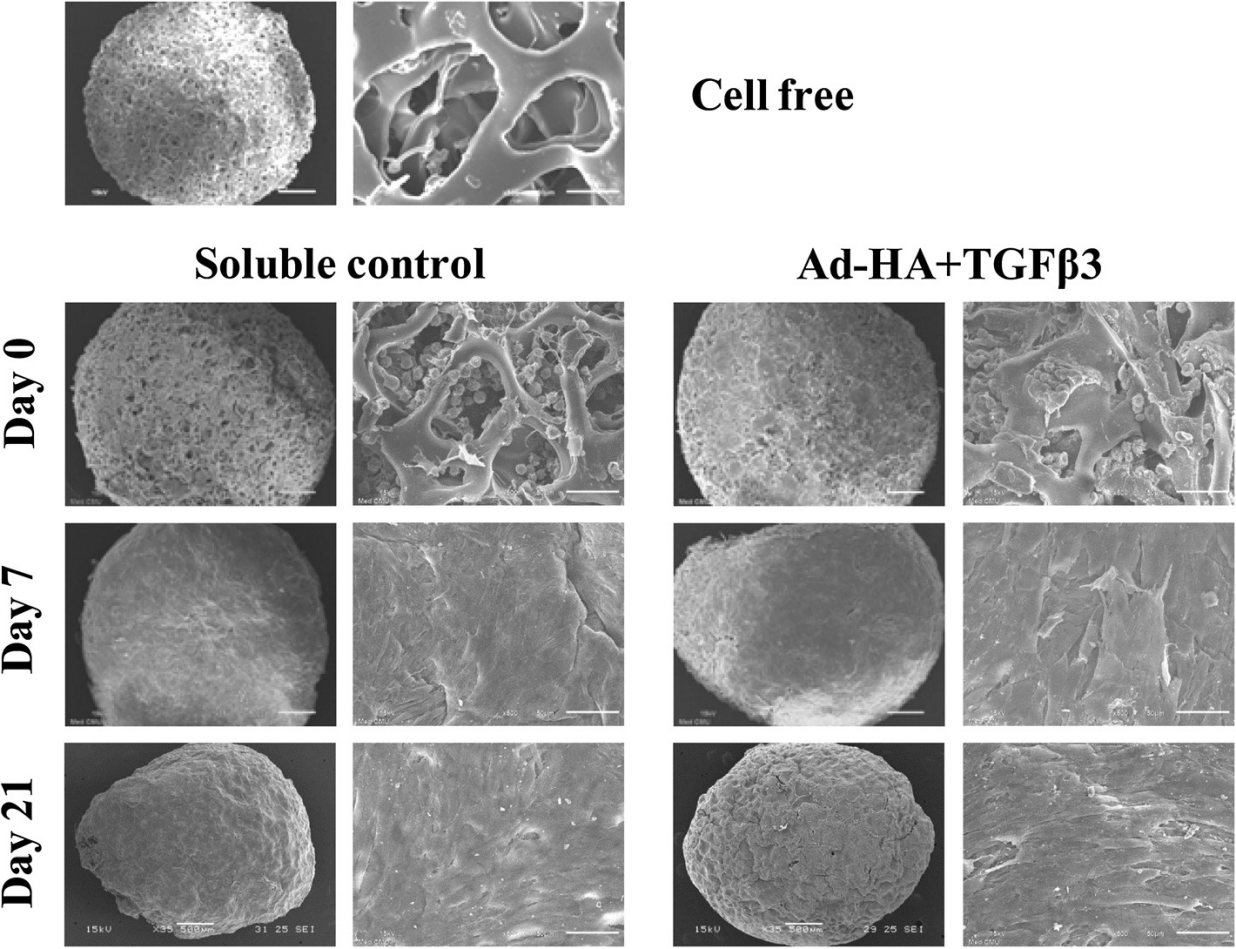
**Scanning electron microscopic examination of chondrocyte-seeded gelatin scaffolds.** A Scanning electron microscope was used on the surface of the cell-free gelatin scaffolds, chondrocyte-cultured HA + TGF-β3 adsorbed scaffolds, and soluble control scaffolds on days 0, 7 and 21 of cultivation. Images are at 35x and 500x insets.

To assess cell morphology and cell distribution further, a scanning EM was used on scaffolds at the end of the 21-day cultivation. This examination showed that there was a complete filling of the extracellular matrix formation in the pores of both soluble control and HA + TGF-β3 adsorbed scaffolds (Figure 6). The surface of HA + TGF-β3 adsorbed scaffolds were also covered with the chondrocytes embedded in extracellular matrix. The chondrocytes showed a spherical morphology comparable with a previous study [25]. The surface of the soluble control scaffolds was smoother than that of HA + TGF-β3 adsorbed scaffolds, and the distinct cell morphology could not be observed.

### TGF-β3 released over 21 days in culture media

**Figure 7:**
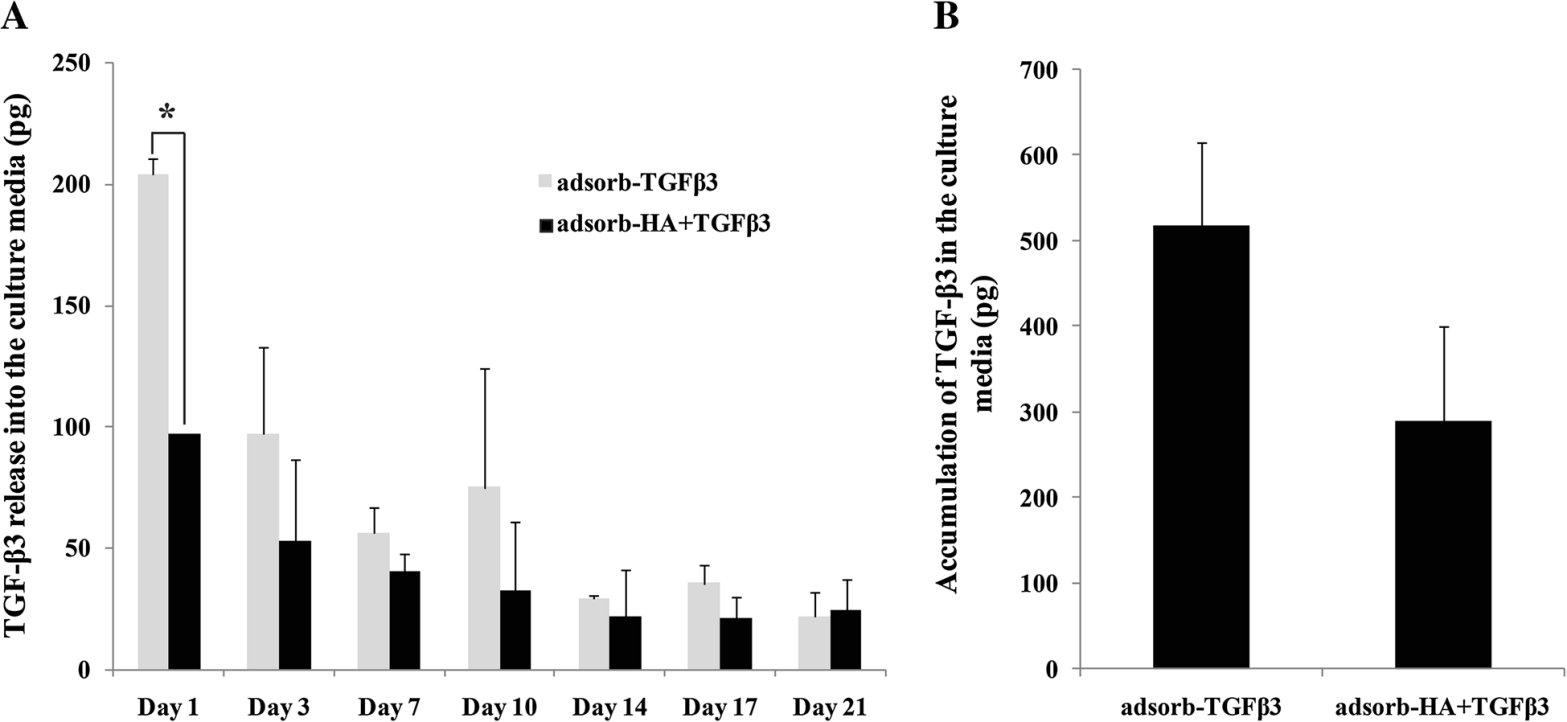
**TGF-β3 release analysis in culture media of chondrocyte-seeded TGF-β3 adsorbed scaffolds and HA + TGF-β3 adsorbed scaffolds.** The amounts of TGF-β3 in culture media on days 1, 3, 7, 10, 14, 17 and 21 of cultivation **(A)** and its accumulation **(B)** were examined. This data represents three separate experiments (*n* = 3). *p-values are < 0.05.

The release of TGF-β3 in culture media of TGF-β3 and HA + TGF-β3 adsorbed scaffolds was quantified and compared. Figure 7A shows that the amount of TGF-β3 released from the HA + TGF-β3 adsorbed scaffolds was lower than from the TGF-β3 adsorbed scaffolds at day 1. Furthermore, the accumulated amount of TGF-β3 released into the culture media of HA + TGF-β3 adsorbed scaffolds was lower than that of the TGF-β3 adsorbed scaffolds (518.21 ± 98.15 *vs* 289.98 ± 110.00 pg) (Figure 7B). The results suggested that the gelatin scaffolds retained much of the TGF-β3 over the 21 days because the amount released was much less than 5 percent of the amount of TGF-β3 used in the adsorbed solution (10,000 pg).

## Discussion

Gelatin is a natural polymer that has been used in cartilage tissue engineering and for cell delivery in cartilage regeneration [26-29]. It is derived from collagen and is suitable because of its lack of antigenicity and retainment of some informational signals, such as the RDG sequence, which can promote cell adhesion, differentiation, proliferation [30,31], and ECM deposition [32]. Absorbable haemostatic porcine gelatin scaffolds, commercially available as SPONGOSTAN®, were obtained from Johnson & Johnson (Denmark). They are medical devices used for haemostatic surgical procedures. They are recommended as being a suitable 3D matrix for chondrocytes [1,22,33] in order to reduce dedifferentiation of the cells *in vitro* and to stabilize the chondrocyte phenotype.

Even though HA-adsorbed gelatin (SPONGOSTAN®) scaffolds [1] and TGF-β3 supplementation in chondrogenic media [22] have previously been investigated for cartilage tissue engineering, this study worked on the new development of combining the effect of both adsorbed exogenous HA and TGF-β3 on chondrocytes in a 3D culture model. This study demonstrated for the first time that the combination of HA and TGF-β3 adsorbed on gelatin scaffolds enhanced the extracellular matrix production and chondrogenic specific gene markers of the embedded primary human chondrocytes.

In this work, gelatin scaffolds were used to mimic a natural extracellular matrix by adsorbing to it the key biomolecules, HA and TGF-β3. This provides a novel factor delivery technique, the adsorption method. The gelatin scaffolds have the ability to enhance cell adhesion and growth by sustaining the release of growth factors [34], which help to simplify the cartilage tissue regeneration process. Moreover, the amount of the stimulating factors and thereby the costs involved become 80% less by using adsorbed scaffold cultures over conventional media supplements.

Previous studies on TGF-β3 suggest that a dose of 10 ng seems to be sufficient to enhance chondrogenesis in 2D and 3D *in vitro* cultures [35-38]. A scaffold control was performed to determine the amount of HA and TGF-β3 released. The data showed that after day 21 of cultivation, the amount of TGF-β3 remained at more than 90 percent of the dose used in the scaffold adsorption process (10 ng).

Gelatin scaffolds were evaluated for 3D chondrocyte cultures for *in vitro* hyaline cartilage tissue regeneration, which used primary human articular chondrocytes. The cell proliferation, extracellular matrix biomolecule content, and histological analysis were designed to determine the effects of HA and TGF-β3 adsorbed gelatin scaffolds on chondrocyte ECM synthesis over the course of the 21-day cultivation. Primarily, at the early stage of cultivation, there was some decline in cell numbers as the cells adapted to being on the matrix, but in comparison with the soluble control scaffolds, the combined adsorbed scaffolds provided the highest cell retention rates (days 1–7). At later stages of cultivation, HA + TGF-β3 adsorbed scaffolds tended to increase in cell proliferation more than other types (days 10–21), whereas the proliferation of cells in soluble control and HA adsorbed scaffolds were lower. These results correspond with previous studies that showed HA to effectively promote chondrocyte proliferation [8]. Because HA is a major component of ECM produced by chondrocytes, it provides an appropriate environment for cell attachment and cell proliferation. Chondrocytes bind to HA via CD44-HA receptor interactions and activate CD44 signaling pathways that play a role in the proliferation of chondrocytes [8]. Although previous studies [39] show that TGF-β3 stimulates cell proliferation, this study, in contrast, found that TGF-β3 adsorbed scaffolds could not promote cell proliferation as well as control scaffolds (Figure 1). However, HA + TGF-β3 adsorbed and soluble control scaffolds had higher cell proliferation rates than HA adsorbed scaffolds. These observations indicated that cell attachment via HA may provide better access to TGF-β3, thus allowing TGF-β3 to enhance HA function, and possibly, HA might retain TGF-β3 on the scaffold which enhances chondrocyte functions. These results correlated with the H&E staining that took place after the 21-day cultivation (Figure 5A). The staining showed large numbers of cells in the pores and on the surface of HA + TGF-β3 adsorbed scaffolds. In cartilage tissue engineering, cell proliferation (viability) is important to achieve tissue regeneration [2-4]. Since chondrocytes were used as the primary cells in this study, any small increase in their proliferation might be meaningful data for ECM production.

This study demonstrated that chondrocytes seeded in the HA + TGF-β3 adsorbed scaffolds expressed chondrogenic-specific extracellular matrix biomolecules, including HA, collagen, and uronic acid more than chondrocytes cultured in other types of gelatin scaffolds. HA not only promotes cell adhesion and proliferation, but also provides positive effects on chondrocyte differentiation [1,8,12]. In addition, TGF-β3 can induce cell differentiation by stimulating the expression of anabolic chondrogenic gene markers, leading to extracellular matrix production [9,10]. According to the Safranin O and Collagen type II staining (Figures 5B and C), HA and TGF-β3 enhance extracellular matrix production.

The expression of chondrogenic genes on days 7, 14 and 21 of cultivation were also examined. On day 7, chondrocytes cultured in HA, HA + TGF-β3 adsorbed, and soluble control scaffolds expressed higher levels of *SOX9* and *ACAN* than those cultured in PBS control. And on day 14, the decreased expressions of these two genes were observed. The *SOX9* transcription factor plays a role in the induction and retainment of the chondrogenic phenotype and cartilaginous matrix formation [40]. It is also known to regulate the expression of *ACAN* [41] and *COL2A1* [42] genes, which encode important cartilage-specific matrix proteins. Nevertheless, both chondrocytes seeded in all of the adsorbed gelatin scaffolds and in the soluble control scaffolds showed an increase in the amount of *COL2A1* expression on day 21, which occurred after the decrease of the *SOX9* expression on day 14. These results indicate that *COL2A1* is under the regulation of other factors besides *SOX9*. On days 14 and 21 of cultivation, the chondrocytes that were seeded in all of the adsorbed scaffolds and in the soluble control expressed higher levels of *COL2A1* expression than the chondrocytes in the PBS control group, and moreover, chondrocytes cultured in HA + TGF-β3 adsorbed scaffold groups showed the highest expression overall. A ratio of *COL2A1* to *COL1A2* is generally used for assessment of chondrocyte phenotypic stability. The *COL2A1*/*COL1A2* ratio of chondrocytes seeded in HA + TGF-β3 adsorbed scaffolds and in the soluble control was significantly higher than the ratio in the PBS control group on day 14. On day 21 of cultivation, the *COL2A1*/*COL1A2* ratio of chondrocytes cultured in HA + TGF-β3 adsorbed scaffolds was higher than in the soluble control group. The previous studies demonstrated that type II collagen gene expression level in native cartilage was markedly higher than in cultured chondrocytes, besides type I collagen gene expression was markedly increased in culture compared to intact cartilage [43,44]. Moreover, they found that type II collagen gene expression exhibited decrease during monolayer cultivation [43]. These evidences indicated that the *COL2A1/COL1A2* ratio was extremely low in chondrocyte cultivation when compares to intact cartilage. Corresponding to our study, the *COL2A1*/*COL1A2* ratio of the cultured chondrocytes was very low in monolayer culture and it was elevated by cultivation in scaffold, however, still lower than in intact cartilage.

These observations suggest that the HA + TGF-β3 adsorbed gelatin scaffolds support dedifferentiation less than and enhance re-differentiation more than the chondrocytes in the monolayer cultures, and therefore, it indicates that they have potential to stabilize the chondrocyte phenotype. These results also indicate that HA and TGF-β3 have anabolic effects on chondrocytes as presented in previous studies [8,23]. They have these effects by promoting the expression of chondrogenic gene markers. Interestingly, HA and TGF-β3 showed a synergistic effect on the retention of chondrogenic properties including enhanced anabolic chondrogenic gene expressions and cartilage matrix proteins production, including HA, proteoglycan, collagen, and uronic acid.

The soluble control scaffolds induced chondrocyte proliferation, ECM production, and chondrogenic anabolic gene expression only during the early period of cultivation. Moreover, these scaffolds showed large amounts of cell proliferation only on the surface of the scaffolds (Figure 5A) but not in the pores of the scaffolds. These observations indicate that soluble HA and TGF-β3 mainly enhance cell proliferation and ECM production of chondrocytes around surface of scaffold, whereas HA + TGF-β3 adsorbed scaffold may induce those in both surface and inside pore of scaffold. Secondly, because the ECM was abundantly produced and tightly covered the scaffolds, TGF-β3 and HA were not able to pass through the ECM layer in order to maintain the chondrocyte function inside the scaffolds.

Furthermore, there was evidence that HA + TGF-β3 adsorbed scaffolds had higher rates of TGF-β3 retainment than those of TGF-β3 adsorbed scaffolds. Thus, HA in HA + TGF-β3 adsorbed scaffolds may be what retains the growth factor TGF-β3 within the scaffolds and helps present the fiber network of HA and TGF-β3 in order to interact and enhance the chondrocyte functions [45]. Evidence suggests that *in vivo* TGF-β3 has a short half-life and may be degraded by proteases and elastases at sites of inflammation [46]. It is possible that TGF-β3 in the adsorbed scaffolds might be degraded after 21 days. However, in this study, HA and TGF-β3 adsorbed scaffolds showed more efficiency in supporting chondrocyte functions and cartilage matrix regeneration than soluble control scaffolds, which were supplemented with fresh HA and TGF-β3. HA is glycosaminoglycan, it has viscous properties, is highly hydrated, and is non-immunogenic. Accordingly, HA has an important physical function. HA maintains the viscosity of interstitial fluid in connective tissue [47], controls water transport [48], and provides protective physiochemical functions. Exogenous HA suppresses the production and activity of matrix metalloproteinases and pro-inflammatory mediators. Therefore, HA has been explored as a drug delivery agent for several target tissues [49]. This study supports the idea that HA could help retain TGF-β3 as a drug delivery agent in gelatin scaffolds.

Many previous studies have reported the comparison between 2D and 3D culture methods on chondrogenic phenotype and cartilage tissue regeneration [18-21,50]. 2D monolayer cultivation has not maintained chondrogenic differentiation gene markers, including *SOX9*, *ACAN*, and *COL2A1*. This corresponds with a lower expression of cartilage extracellular matrix than what is seen in 3D cultivation. In contrast, 3D cultivation is accepted as the way to help maintain the chondrocyte phenotype by increasing both the chondrogenic mRNA and extracellular matrix expression. Scaffolds provide a 3D environment in which cell-cell and cell-extracellular matrix interactions are promoted, and they are likely to be an important factor for chondrocyte functions in cartilage regeneration.

## Conclusion

Chondrocyte dedifferentiation occurs in routine cultivation of 2D monolayer culture, and 3D culture can be used to retain chondrogenic phenotypes. In this study, a functional scaffold was developed based on a novel strategy that enhances redifferentiation and promotes matrix production in 3D culture. An investigation into this scaffold design has shown that HA + TGF-β3 adsorbed scaffolds are suitable for supporting chondrocyte expansion and the enhancement of extracellular matrix production, including HA, s-GAG, uronic acid, and collagen. In addition, this functional scaffold gives improved expression of anabolic chondrogenic genes, including *SOX9*, *ACAN*, and *COL2A1*, when compared to the control groups. Since HA and TGF-β3 show a synergistic effect on the induction of chondrogenic properties when adsorbed onto HA + TGF-β3 adsorbed gelatin scaffolds, it is believed that these scaffolds may offer advantages as regenerative cartilage tissue models for *in vitro* 3D human chondrocyte culture.

## Methods

### Human articular chondrocyte preparation

Human articular cartilage from non-osteoarthritis joints were collected during notchplasty operations from the articular knee cartilage of 18-45 year old patients at Maharaj Nakorn Chiang Mai Hospital, Chiang Mai, Thailand. Chondrocytes were isolated and purified by standard protocols as described below. All patients gave consent, and all procedures of this study were approved by the Research Ethical Committee, Faculty of Medicine, Chiang Mai University, Thailand (the ethics approval code was 070CT111016).

Cartilage was carefully cut away from the underlying bone, and the chondrocytes were isolated by a collagenase digestion and then grown as monolayers in Dulbecco’s Modified Eagles medium (DMEM) containing 10% (v/v) fetal calf serum (FCS), 100 U/ml penicillin and 100 μg/ml streptomycin until an 80% confluency was reached. The cells were cultured in a humidified incubator with 5% CO_2_ at 37°C. DMEM, penicillin, streptomycin, and FCS were purchased from Gibco (USA). Collagenase was obtained from Calbiochem (Germany).

### Preparation of HA/TGF-β3 was physically immobilized on a gelatin scaffold by the adsorption method

The gelatin scaffolds SPONGOSTAN® were obtained from Johnson & Johnson. Circular gelatin scaffolds were cut using a metal borer or punch with a diameter of 5 mm. They were sterilized by autoclave at 121°C, 15 pounds and 25 minutes. For the adsorption process, the scaffolds were transferred to a sterile 24-wells tissue culture plate and incubated with 100 μl solution of (a) 0.07% (w/v) HA (Viscoseal) in PBS (adsorb-HA), (b) 100 ng/ml TGF-β3 (Peprotech, USA) in PBS (adsorb-TGF-β3) or (c) 0.07% (w/v) HA and 100 ng/ml TGF-β3 in PBS (adsorb-HA + TGF-β3) and incubated at 37°C for 4 hours. A control scaffold was adsorbed with PBS alone in this step. For the soluble control group, the scaffold was processed the same as the PBS control group, and soluble 0.07% (w/v) HA and 10 ng/ml TGF-β3 were added directly into culture media each time the media was changed.

### Cell seeding and culture conditions

The primary human chondrocytes were expanded through 4 passages (P4), and cells were harvested at 80% confluency with 0.25% trypsin and 2.5 mM EDTA. The scaffolds were transferred to a sterile 24-wells tissue culture plate and then 20 μl of 25×10^6^ cells/ml cell suspensions (500,000 cells/scaffold) were directly seeded onto the scaffold and left to attach at 37°C and 5% CO_2_ for 4 hours. After attachment, 1 ml of DMEM with 10% FCS was added and incubated at 37°C under 5% CO_2_ overnight. The next day (day 0), the scaffolds were transferred to new culture wells, and 1 ml of chondrogenic media (DMEM, 10% FCS, 10^−7^ M dexamethasone, 25 μg/ml ascorbic acid (Sigma, USA) and 1x insulin-transferrin-selenite (ITS) supplement (Gibco, USA)) were added, and then they were incubated at 37°C under 5% CO_2_ for up to 21 days. Culture media was changed and kept at 3-day intervals for HA release analysis. Cells cultured in scaffolds on day 21 were digested at 60°C overnight with 2 units of papain. Papain digests were analyzed for cartilage extracellular matrix anabolism by determining the content amount of HA, s-GAG, collagen, and uronic acid. Additionally, the culture media were assayed for the release of TGF-β3 growth factor.

### Scaffold controls

For the scaffold controls, the scaffolds were cultured in chondrogenic media without cells and incubated at 37°C under 5% CO_2_. Culture media was changed and kept at 3-day intervals for HA and TGF-β3 release analysis using enzyme linked immunosorbent assay (ELISA), and was used as an indirect method to measure the levels at which HA and TGF-β3 were adsorbed to the scaffold. The amount of HA and TGF-β3 remaining on the scaffold were determined by subtracting the amount of released HA and TGF-β3 in the culture media from the original amount used in the adsorbed solution. This method was necessary because the amount of HA and TGF-β3 adsorbed onto the scaffold could not be detected by ELISA. After 21 days, the scaffold controls were digested by papain and analyzed for HA content in order to normalize it with the production values of the cell-seeded scaffolds.

### Cell proliferation assay

Cell viability or proliferation was assessed by Alamar Blue assay, following the supplier’s instructions [51]. Samples of the chondrocyte-seeded scaffolds were daily incubated in media supplemented with 10% (v/v) Alamar Blue fluorescent dye (Sigma, USA) for 4 hours at 37°C. The adsorbance was measured at 570 and 600 nm by a micro-plate reader spectrophotometer**.**

### Assay of extracellular matrix (ECM) biomolecules

#### Hyaluronic acid

Hyaluronic acid which was released into the culture media and then accumulated on the scaffolds was examined using a competitive inhibition-based, enzyme-linked immunosorbent assay (ELISA) for HA as previously described [52]. Briefly, the samples or standards of HA (Healon®) at various concentrations (19–10,000 ng/ml) were incubated with biotinated hyaluronan-binding proteins (B-HABPs) (1:200), diluted in 0.05 M Tris–HCl buffer, pH 8.6 at room temperature for 1 hour, and then added to the microplate, which was pre-coated with umbilical cord HA overnight at 4°C (100 μl/well of 1 mg/ml HA) and blocked with 1% BSA (150 μl/well) for 1 hour at room temperature. The wells were subsequently washed and 100 μl of peroxidase-conjugated anti-biotin antibodies (1:2,000 dilute in PBS) were added and incubated at room temperature for 1 hour. The wells were subsequently washed and 100 μl of peroxidase substrate were added and incubated at room temperature for 10 minutes to allow the color to develop. The reaction was stopped by using 50 μl/well of 4 M sulfuric acid, and the absorbance was determined using a microplate reader spectrophotometer at 492/690 nm. The concentration of HA in the samples was calculated with reference to a standard curve.

#### s-GAG

The assay is based on a metachromatic shift in absorption maximal from 690 nm to 535 nm as a complex compound is formed in the mixture of 1,9-dimethylmethylene blue (DMMB) and the sulfated glycosaminoglycan (s-GAG) in the samples and then compared to standard chondroitin 6-sulfate (CS-C: shark cartilage extract) [53]. The DMMB solution was added to CS-C standards, culture media samples, or papain digest solutions, and then the absorbance values at 520 nm were measured with a microplate reader spectrophotometer.

#### Collagen content determined by hydroxyproline assay

Papain-digested samples were hydrolyzed with 6 N HCl for 24 hours at 60°C and neutralized with 12 N NaOH. The samples or hydroxyproline standards were reacted with freshly prepared oxidizing solution (Chloramine-T) and further incubated for 5 minutes at room temperature. After incubation, Ehrlich’s reagent was added, and the reaction mixtures were incubated for 45–60 minutes at 60°C. The absorbance was measured at 570 nm by a microplate reader spectrophotometer.

#### Uronic acid content determined by carbazole reaction

The uronic acid content in the scaffolds was analyzed according to the release of the monosaccharide from glycosaminoglycan in ECM by acid hydrolysis, and the reaction with the carbazole reagent was detected at the maximal adsorption at 540 nm in a microplate reader spectrophotometer [54]. The papain-digested samples or glucuronic acid standards were hydrolyzed with the sulfuric acid-borate reagent at 100°C for 15 minutes and cooled down in an ice bath. The carbazole reagent was added, and the reaction took place at 100°C for 15 minutes and then was cooled down in an ice bath. The absorbance was measured at 540 nm by a microplate reader spectrophotometer.

#### DNA content

The values of HA, s-GAG, collagen and uronic acid production were normalized per cell with the DNA content. Hoechst 33258 dye or bisbenzimide is a fluorescent dye that binds to AT-rich regions of DNA, allowing it to be used as a way to detect the quantities of DNA. Hoechst 33258 dye (Sigma, USA) is excited by ultraviolet light at around 356 nm and emits a blue/cyan fluorescence light with an emission wavelength of 465 nm [55]. Ten μL of papain-digested samples or DNA standards from calf thymus were diluted in 2 ml of dye/TNE buffer solution, and the fluorescence of the samples was evaluated as described above.

### Chondrogenic anabolic gene expressions by quantitative real-time RT-PCR

Total RNA from cultured scaffolds on days 7, 14, and 21 were extracted using an RNA extraction kit (GE Healthcare, UK), in accordance with the manufacturer’s protocol. 200 ng of total RNA were converted to cDNA using RevertAid™ H minus first strand cDNA synthesis kit (Fermentus Life science, USA). In order to determine the chondrogenic gene expression, SYBR Green detection was used, and the values were normalized using *glyceraldehyde-3-phosphate dehydrogenase (GAPDH)*. A real-time quantitative polymerase chain reaction (PCR) was performed in a DNA Engine (ABi 7500) using SYBR GREENER qPCR UNIVERSAL (Invitrogen, USA). Primers sequences are as follows: *Aggrecan core protein (ACAN)* forward: 5′CTGTTCAGGGACAGAATGTGCT3′*, Aggrecan core protein (ACAN)* reverse: 5′TCGATATGCTTCACAGTTCTAGGG3′*, Collagen type I (COL1A2)* forward: 5′TTTTGGCCATCTCTTCCTTCA3′*, Collagen type I (COL1A2)* reverse: 5′TGTGGATGCCTC TTGGGTATC3′*, Collagen type II (COL2A1)* forward: 5′TCCTCTTCTTGAGCTGGACTCATTCT3′*, Collagen type II (COL2A1)* reverse: 5′CGCTCTGCAAACTGGAGGTC3′*, SOX9* forward: 5′ GAAGGTGAAGGTCGGAGTC3′*, SOX9* reverse: 5′GAAGATGGTGATGGGATTTC3′*, GAPDH* forward: 5′GAAGGTGAAGGTCGGAGTC3′*, GAPDH* reverse: 5′ GAAGATGGTGATGGGATTTC3′. Relative expression levels of each gene were calculated by normalizing them with the expression of *GAPDH* by the 2^*-ΔCT*^ method [56].

### Histological immunohistochemistry

#### Scaffold section preparation

The cells cultured on the scaffolds for 21 days were fixed in a solution of 4% paraformaldehyde and dehydrated through a graded ethanol series including 50%, 70%, 95%, 100% ethanol and xylene in the last step. Then the cultured scaffolds were embedded in paraffin wax, casted into blocks, and sectioned at a thickness of 5 μm [17].

#### Haematoxylin-eosin (H&E) staining

The sections were deparaffinized with xylene and rehydrated through a graded ethanol series including 95% and 70% ethanol and PBS in the last step. The sections were stained in a hematoxylin solution and counterstained with eosin. The stained sections were dehydrated through a graded ethanol series and xylene and were then mounted using mounting media.

#### Safranin O staining

Sections were stained with safranin O. This staining process is used for the detection of cartilagesulfated glycosaminoglycan as well as mast cell granules on paraffin-embedded tissue sections. The sections were deparaffinized, rehydrated, and stained with safranin O for 30 minutes. The stained sections were dehydrated and mounted [57].

#### Immunohistochemistry for collagen type II

In order to perform an immunohistological analysis of collagen type II synthesis, after the sections were deparaffinized and rehydrated, the sections were digested with 0.25% trypsin, 1.45 IU/ml testicular hyaluronidase and 0.25 IU/ml chondroitinase ABC for 15 minutes at 37°C in each step, respectively. Then the sections were blocked for the endogenous peroxidase with 3% H_2_O_2_ in PBS, and the non-specific sites were blocked with 3% BSA in PBS. The sections were incubated with mouse monoclonal anti-collagen type II (1:100) for overnight at 4°C, followed by incubation with anti-mouse polyvalent immunoglobulin conjugate HRP (1:25) for 1 hour at room temperature. After washing with PBS, DAB solutions were added as previously described [58].

### Scanning electron microscopic examination

On days 0, 7 and 21, cultured scaffold samples were washed twice with PBS and fixed in 0.1 M of phosphate-buffer solution containing 3% glutaraldehyde overnight at 4°C. Samples were dehydrated through a graded series of ethanol and acetone until they reached their critical point of drying using a CPD 7501 (critical point drier) and then were mounted on stubs and sputtered with an ultrathin layer of gold in a SEM-coating system using SPI-MODULE^TM^ Sputter Coater. Specimens were studied using a JEOL 5910 LV SEM apparatus at an accelerating voltage of 15 kV.

### Quantification of TGF-β3

The amount of released TGF-β3 was quantified in the culture media using the sandwich enzyme-linked immunosorbant assay (ELISA) kit (Human TGF-β3 ELISA kit, Koma biotech), in accordance with the manufacturer’s instructions.

### Statistic analysis

Significance of differences between the groups of data was determined using a one-way analysis of variance (one-way ANOVA) test, and all groups were expressed as mean ± SD. Statistical significance was assumed at *p < 0.05*. The data represents three separate experiments (*n* = 3).
